# 

**Published:** 1894-04-07

**Authors:** 


### CONTENTS.

A Teaching University for London - - - - - - 1

Around the Hospitals - - - - - - - - - - 2

On Modern Progress in Ophthalmic Medicine and Surgery. By Robert Brudenell Carter - - - - 3

Studies in Applied Sociology. VI.—Social Pathology and Education - - - - - - - - - - - - - 4

Consumption and Infection - - - - - - - - - 5

The Field of Science - - - - - - - - - - - 6

Annotations.—Physiology and Property—Professor Foster and Scientific Pin-Pointers—Do Doctors Feel? - - - - - - 7

Notes and News - - - - - - - - - - - - - - 8

The International Medical Congress at Rome - - - 15

Medical Progress and Hospital Clinics—

Electricity and Its Use in Medicine. I. - - - - - - 9

Medical Progress—Small-pox - - - - - - - - 11

Pneumonia - - - - - - - - - - - 12

Progress in Surgery—Rectal Surgery - - - - - - 14

The Institutional Workshop—

Foreign Hospital Intelligence - - - - - - - 17

The Irish Lunacy Blue Book - - - - - - - - 17

Practical Departments - - - - - - - - - - 18

The Story of the Insane from Year to Year - - - 19

Construction Notes—Hospital Festivals, Meetings, &c. - 19

Scraps and Gleanings - - - - - - - - - - 20

### EXTRA SUPPLEMENT.—THE NURSING MIRROR.

News from the Nursing World.—Princess Christian at Oxford—A New Nurses' Library—Trained Nurses' Annuity Fund—Newton Abbot—District Nursing in Gloucester—Nurses at Bishop Auckland—Better Prospects for Paupers—A Dangerous Precedent—A New Nurses' Home—Training at Pennsylvania—A Woman's Notion of a Fine—Short Items - - - - i

On General Nursing. VI.—Catarrh - - - - - - - iii

Eastertide in Spain - - - - - - - - - - - iv

Women's Volunteer Medical Staff Corps - - - - - iv

Lectures at St. George's Hospital—The Antiseptic System (continued) - - - - - - - - - - - - - - v

Deaths from Carbolic Acid - - - - - - - - - - v

The Mutual Relations of Nurses and Medical Women. By Mrs. Scharlieb, M.D. - - - - - - - - - - - vi

Clapham School of Midwifery - - - - - - - - - vi

The Nursing of Army Sisters - - - - - - - - - vii

Nursing in the United States - - - - - - - - - vii

Women's Work.—Practical Massage (continued) - - - - viii

Where to Go—Notes and Queries - - - - - - - - viii

The Muses' Looking Glass.—Where to Spend a Holiday—Along the Roman Wall - - - - - - - - - - - - ix

The New York Teachers' College - - - - - - - - ix

Everybody's Opinion - - - - - - - - - - - - x

Appointment —For Reading to the Sick - - - - - x

